# Extreme pulse dosing of 10 kHz spinal cord stimulation: how low can you go?

**DOI:** 10.3389/fpain.2025.1633424

**Published:** 2025-10-01

**Authors:** Mayank Gupta, Amy Reinert, C. O. West, Rose Province-Azalde, Kerry Bradley

**Affiliations:** ^1^ Neuroscience Research Center, LLC, Overland Park, KS, United States; ^2^ Nevro Corporation, Redwood City, CA, United States

**Keywords:** 10 kHz, chronic low back pain, duty cycling, high-frequency stimulation, pulse dosing, spinal cord stimulation

## Abstract

**Background:**

Pulse dosing of high frequency spinal cord stimulation at 10 kHz (10 kHz SCS) may offer comparable clinical benefits as continuous 10 kHz SCS, but extreme pulse dosing (EPD) has not been studied.

**Methods:**

Patients using an implantable pulse generator (IPG) with 10 kHz SCS to treat chronic back or leg pain were enrolled. After baseline assessments, patients underwent “EPD titration” starting at an EPD setting of 3%. Patients who preferred the EPD tried progressively lower EPD settings (0.6%, 0.3%, 0.14%, 0.06%), each for 7–10 days, until reaching an EPD they did not prefer over that previously tried. Patients were then followed up for 3 months at their lowest preferred EPD. All study visits included assessment of adverse events and patient-reported outcomes, including the numeric rating score (NRS) for pain intensity, Patient Global Impression of Change (PGIC), Oswestry Disability Index (ODI), and the PROMIS-SF for sleep disturbance. Device charging information was uploaded from the IPG at each visit.

**Results:**

Eighteen patients completed testing (13 M/5 F; mean age, 61 years); 14 patients (78%) reporting a preferred EPD (at any setting) to standard 10 kHz SCS. Among 18 patients, the most common lowest preferred EPD was 0.14% (28%), followed by 0.06% (22%) and 3% (17%). All post-SCS pain scores were lower than pre-SCS pain scores (median NRS, 8.5 vs. 3.0; *p* = .004). For overall pain, NRS values did not vary significantly across timepoints after the pre-SCS period (median range, 3.0–4.0; *p* > .05). Similarly, patient satisfaction, PGIC, ODI, PCS, and PROMIS-SF scores for EPD did not vary significantly from those at baseline. Daily IPG recharge times were significantly shorter using the patient's lowest preferred EPD than at baseline (median minutes, 3.0 vs. 31.8; *p* = .0001).

**Conclusions:**

EPD 10 kHz SCS may offer the same pain relief and quality-of-life benefits as standard 10 kHz SCS, while reducing recharge requirements and potentially lowering the risk of therapy habituation.

## 1 Introduction

Chronic lower back pain affects about 10% of adults in the United States and over 8% of adults globally (1). This condition is often not amenable to standard therapies, including analgesic treatments or back surgery, contributing to the complexity of its management as well as high care costs, estimated to total about 40 billion dollars annually in the United States (1, 2). As an effective, non-pharmacological treatment for pain, high frequency spinal cord stimulation at 10 kHz (10 kHz SCS) provides an alternative approach for the management of chronic back pain. Evidence from randomized clinical trials of patients with chronic back pain indicate that, compared with paresthesia-based low frequency SCS, 10 kHz SCS provides significantly more pain relief and improvement in quality-of-life, with effects durable up to 24 months (3–5). Indeed, evidence suggests that neurons involved in inhibiting chronic pain (dorsal horn GABAergic neurons) may be selectively driven by 10 kHz SCS but not by SCS of lower frequencies (6).

Notably, all SCS therapies, whether low frequency or 10 kHz SCS, are usually programmed to deliver continuous stimulation. While continuously-delivered 10 kHz SCS may require more device recharging than lower frequency SCS systems, patients have reported similar or better satisfaction with their device charging experience using the 10 kHz SCS system (7). Nevertheless, the greater demand for energy with continuous 10 kHz SCS could affect the convenience to patients provided by rechargeable implantable pulse generators (IPGs), a device introduced in the early 2000s that eliminates the need for frequent re-implantation or wearable external power sources (8). Furthermore, it has been hypothesized that continuous activation of neurons may promote central nervous system adaptation over time, leading to reduced therapeutic efficacy in some patients (9).

In this context, interest has grown in the idea of pulse dosing (PD) to deliver 10 kHz SCS at regular intervals, reducing device recharge times and potentially extending the long-term durability of pain control (10). However, few studies have evaluated this issue and data remain sparse regarding the lowest interval duration needed for PD to remain effective in reducing chronic lower back pain. To help address this knowledge gap, we evaluated the effect of extreme pulse dosing (EPD) with 10 kHz SCS on patient outcomes, using PD settings up to 50 times lower than the smallest studied PD values.

## 2 Methods

### 2.1 Study design

This study was a prospective case series of patients being treated for chronic back or leg pain with 10 kHz SCS delivered via the SENZA SCS IPG (the SENZA SCS IPG is fully-implantable, electrical pulse generator consisting of a rechargeable battery and electronics which are hermetically sealed within a titanium can; the IPG is fully programmable via two-way transcutaneous radiofrequency telemetry; to deliver stimulation, the IPG is connected to two thin catheter-type “leads” each containing eight platinum-iridium electrode contacts, which are positioned in the dorsal epidural space over the low thoracic spinal segment to deliver the electrical stimulation to the spinal cord; the IPG itself is implanted in the upper buttock region). Investigational review board approval was obtained prior to subject enrollment (WCG Investigational Review Board Study Number: 1309273, 5/26/2021) and all subjects provided informed consent. The study was conducted in accordance with local clinical research and data protection regulations, good clinical practice guidelines (ISO14155), and the Declaration of Helsinki. Due to an administrative oversight prior to enrollment, this study was retrospectively registered on clinicaltrials.gov on March 26, 2025 (NCT06897280); *post hoc* study registration is not believed to affect study execution or results, since the study is a post-market evaluation of commercially-available program settings and all subjects were enrolled from a single study center.

**Figure 1:**
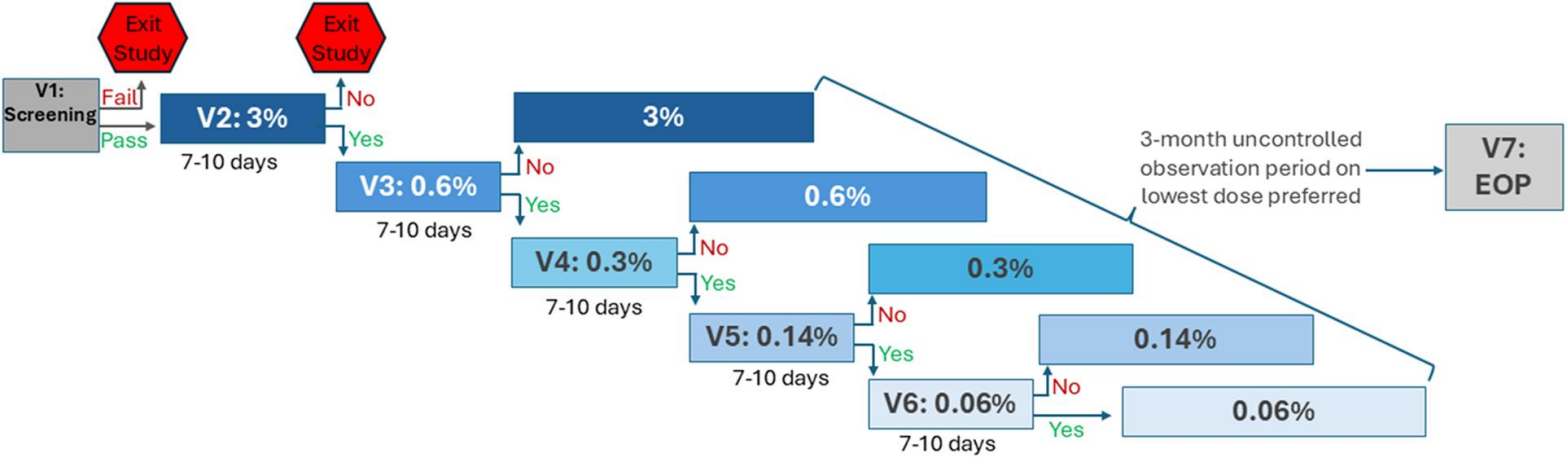
Study design. EOP, end of observation period; PD, pulse dosing; V, visit. Patients who passed eligibility screening (V1) entered the EPD titration phase (V2–V6), in which progressively lower PD settings were assessed sequentially, each for 7–10 days. At V2, PD was set to 3%; patients who did not find equivalent or prefer 3% PD to the baseline setting exited the study. All other patients progressed through 7-to-10-day assessments of lower PD settings (0.6%, 0.3%, 0.14%, 0.06%) until reaching a PD they did not find equivalent or prefer over the previous setting. After the EPD titration phase, patients entered a 3-month observation period on their lowest preferred PD setting. Patients completed the final study visit, V7, at the end of the observation period (EOP).

**Figure 2:**
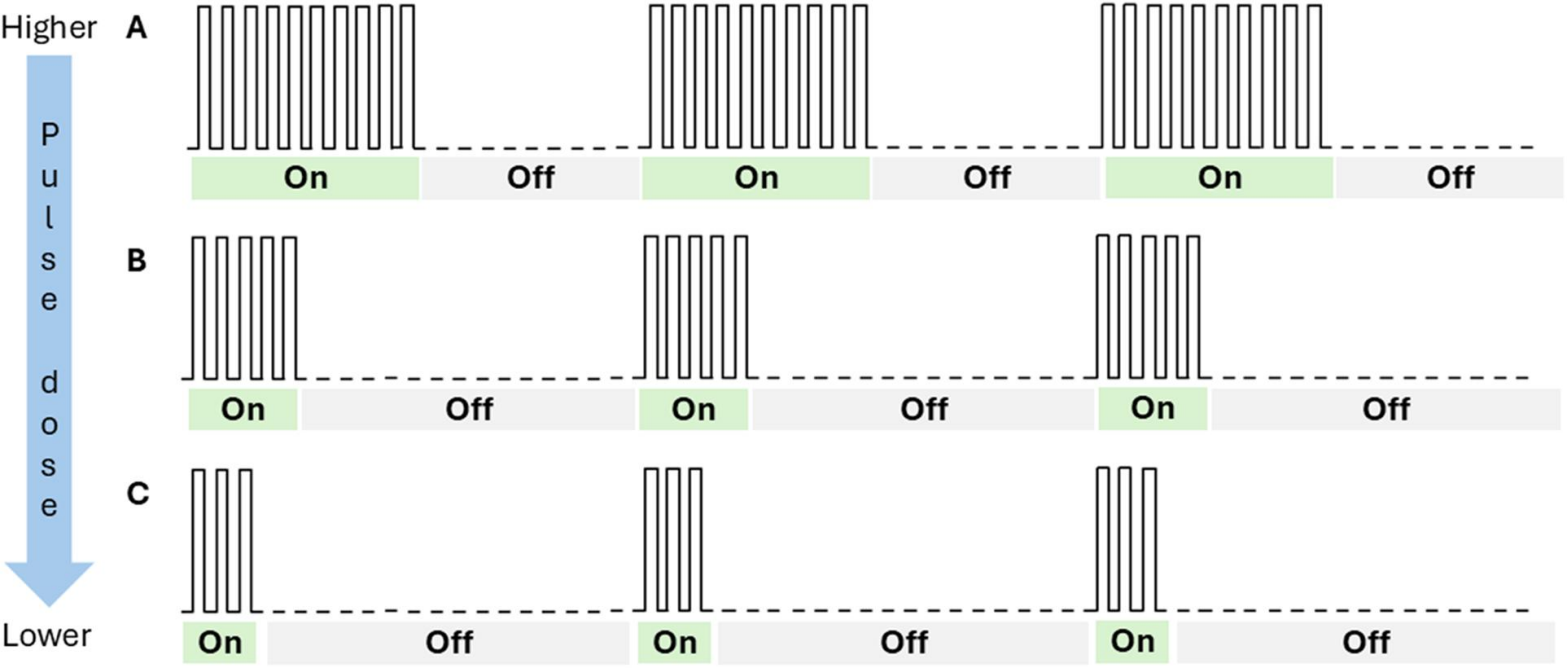
Depiction of pulse dosing settings. This figure is a conceptual drawing and not a representation of actual pulse dose settings. “On” represents periods of electrical stimulation; “Off” represents periods of no stimulation. At higher pulse dose settings, patients receive longer durations of electrical stimulation than at lower pulse dose settings. The duration of “On” and “Off” periods is independent of the frequency or amplitude of the stimulation. In the current study, the “On” periods were delivered at a frequency of 10 kHz. In the figure, panel A would represent a 50% pulse dose setting, while panel B would represent a 25% pulse dose setting, and panel C would represent a 16% pulse dose setting.

After completing eligibility screening, enrolled patients underwent a baseline assessment (Visit 1) before entering the study's EPD titration phase involving successive 7-to-10 day assessment periods of progressively lower PD settings (Visits 2–6; Figure 1). All PD settings used stimulation at a frequency of 10 kHz but varied regarding the duration of delivered electrical stimulation (i.e., SCS is “On”; Figure 2). PD settings were programmed into the patients' IPG at clinic-based EPD titration visits. For the first EPD titration assessment the IPG was set at a 3% PD setting, implemented as 20 s on and 10 min off. After 7–10 days, patients who reported not finding the 3% PD setting to be as good as or preferable to the baseline 10 kHz SCS exited the study. All other patients proceeded to a 7-to-10-day assessment of the next lowest dose (0.6% PD). This process continued through progressively lower PD settings (0.3%, 0.14%, 0.06%), until the patient reached a PD setting they did not prefer or find as good as the previous PD setting. After reaching their lowest preferred PD setting, patients entered a 3-month observation period. At the end of the observation period, patients returned to the clinic for their final visit (Visit 7).

### 2.2 Patient population

Patient enrollment was conducted from September 2021 through November 2023 at a single clinical site in the United States. Patients receiving 10 kHz SCS treatment for chronic back or leg pain were invited by site staff to participate in the study. Interested patients were screened for eligibility using the following criteria: chronic intractable back pain or leg pain of neuropathic origin, as determined by the physician; has a permanent implant of the 10 kHz SENZA IPG System, implanted at least 3 months before baseline; 18 years of age or older; and willing and capable of giving informed consent. Patients who passed eligibility screening were enrolled in the study.

### 2.3 Outcome measures

The primary outcome assessed in this study was the response rate, defined as the proportion of patients reporting that EPD at any PD setting (i.e., 3%, 0.6%, 0.3%, 0.14%, or 0.06%) was as good as or preferable to their baseline 10 kHz SCS treatment. To further understand the response rate, the distribution of patients' lowest preferred PD setting was also evaluated. In addition, several patient reported outcomes (PROs) were evaluated at the following four time points: pre-SCS therapy, baseline, after the EPD titration assessment period of the patient's lowest preferred dose setting, and at the end of the 3-month observation period (End of OP). Pain intensity was measured using the numerical rating scale (NRS), a visual analogue scale in which 0 indicates no pain and 10 indicates the worst pain imaginable. The pre-SCS NRS score was obtained from the pre-surgical assessment conducted before the patient's 10 kHz SCS implant (i.e., before entering the study). During study follow-up, NRS scores were obtained by averaging the daily values recorded in patient diaries, completed at home during the 7 days before the study visit.

Other PROs were measured at all study visits but not assessed for the pre-SCS therapy period. PROs included responses to questions on patient satisfaction and the Patient Global Impression of Change (PGIC) assessment. The PGIC is a single, self-administered question in which patients used a 7-point scale to evaluate the change in their condition compared with the level at the baseline visit. PGIC responses range from “much worse” to “much improved”. Patients also completed questionnaires for the Oswestry Disability Index (ODI), a 10-item index used worldwide to assess functional disability associated with back pain.10 Total scores for the ODI range from 0 (minimal disability) to 100 (severe disability; e.g., bedridden). To evaluate the frequency of emotional and cognitive responses to pain, patients were administered the Pain Catastrophizing Scale (PCS), a 13-item index widely used in research, including clinical trials, to assess the impact of back pain on patient's well-being (11–14). For each PCS item, responses range from 0 (“not at all”) to 4 (“all the time”), leading to a total score range of 0–52. Self-reported sleep disturbance was assessed using the PROMIS Sleep Disturbance short form (PROMIS-SF 8a), an 8-item survey regarding sleep quality, sleep depth, and sleep-associated restoration with a score range of 0–40 (15). Patients were also administered the Brief Pain Inventory (BPI) short form, a 9-item questionnaire validated for pain assessment in patients with lower back pain (16). The BPI comprises items that assess pain intensity, use of pain treatments, and pain interference with daily life activities and has a total score ranging from 0 to 10. In addition to PROs, the study evaluated IPG charging duration by downloading data directly from the IPG during study visits.

### 2.4 Statistical analysis

Study data were descriptively analyzed to assess frequencies and distributions of patient outcomes. Continuous variables were summarized using medians, ranges, means, and standard deviations. Categorical variables were summarized using frequency distributions. Shapiro–Wilk tests were used to assess the normality of data distributions, which determined the choice of parametric (normal) or non-parametric analyses (non-normal). In particular, to compare patient outcomes across time periods repeated measures (RM) ANOVA tests were used for normally distributed data, and Friedman/Friedman-Nemenyi-Q tests were used for non-normally distributed data.

## 3 Results

### 3.1 Patient characteristics

A total of 22 patients were screened for study eligibility, among whom 2 were excluded, 1 for having a co-existing implantable device and the other for having had an interventional procedure to treat their back/leg pain within the preceding 30 days (Table 1). Thus, the study enrolled 20 patients. Of these, two subjects withdrew consent prior to study completion, yielding 18 patients completing the study. These 18 patients consisted of 13 men and 5 women, with ages ranging from 37 to 76 years old (mean age, 61 years). Most enrolled patients were White (*n* = 16). The most common pain indication among enrolled patients was failed back surgery syndrome with lumbar radiculopathy (*n* = 11), followed by lumbar radiculopathy (*n* = 4). Eight patients presented with a Baseline program that used PD, ranging from 14% to 67%. There was no significant correlation between Baseline PD and chosen EPD (*p* = 0.75). Review of study log files indicated that subjects had Baseline and EPD titration stimulation delivered continuously for over 95% of the duration of each titration evaluation period.

**Table 1:** Characteristics of screened patients.

Subject No.	Screening date	Age, years	Sex	Race/ethnicity^a^	Weight, lb	Height, in	Pain indication	Enrolment status
1	08/09/2021	72	F	White	240	64	Failed back surgery syndrome with lumbar radiculopathy	Failed screen
2	08/09/2021	58	F	White	198	66	Failed back surgery syndrome with lumbar radiculopathy	Failed screen
3	11/16/2021	64	F	White	208	66	Lumbar radiculopathy	Completed
4	01/07/2022	65	M	White	200	70	Failed back surgery syndrome with lumbar radiculopathy	Completed
5	01/17/2022	50	M	Black	254	72	Failed back surgery syndrome with lumbar radiculopathy	Completed
6	02/04/2022	60	M	White	180	64	Lumbar radiculopathy	Completed
7	03/03/2022	58	M	White	240	74	Post laminectomy syndrome with lumbar radiculitis	Completed
8	03/15/2022	49	M	White	315	70	Lumbar radiculopathy with post surgical scar neuralgia left hip	Completed
9	04/12/2022	75	M	White	235	71	Failed back surgery syndrome with lumbar radiculopathy	Completed
10	04/13/2022	72	M	White	224	68	Failed back surgery syndrome with lumbar radiculopathy	Completed
11	04/15/2022	44	F	White	182	62	Failed back surgery syndrome with radiculitis	Completed
12	05/17/2022	64	M	White	186	72	Lumbar radiculopathy	Completed
13	05/23/2022	62	M	White	220	71	Failed back surgery syndrome with lumbar radiculopathy	Completed
14	06/8/2022	76	M	White	210	69	Failed back surgery syndrome with radiculitis	Completed
15	06/24/2022	50	F	White	145	61	Failed back surgery syndrome with lumbar radiculopathy	Completed
16	07/18/2022	58	M	White	185	72	Failed back surgery syndrome with lumbar radiculopathy	Completed
17	06/15/2023	66	F	White	175	64	Failed back surgery syndrome with lumbar radiculopathy	Completed
18	06/22/2023	37	M	Hispanic	145	65	Lumbar radiculopathy with neuropathic pain	Completed
19	07/18/2023	76	M	White	170	71	Lumbar radiculopathy	Completed
20	11/30/2023	75	F	White	162	62	Failed back surgery syndrome with lumbar radiculopathy	Withdrew prior to completion
21	11/29/2023	55	M	White	245	73	Failed back surgery syndrome with lumbar radiculopathy	Withdrew prior to completion
22	11/30/2023	75	F	White	162	62	Lumbar radiculopathy	Completed

F, female; M, male.

Weight is given in pounds. Height is given in inches. Age, weight, and height values reflect measurements taken at screening. Two patients failed screening: one patient had a co-existing implantable device and the other patient had undergone an interventional procedure to treat back/leg pain other than Senza HF10 therapy in the preceding 30 days.

^a^ Black refers to Black or African American.

### 3.2 Extreme pulse dosing response rate

**Figure 3:**
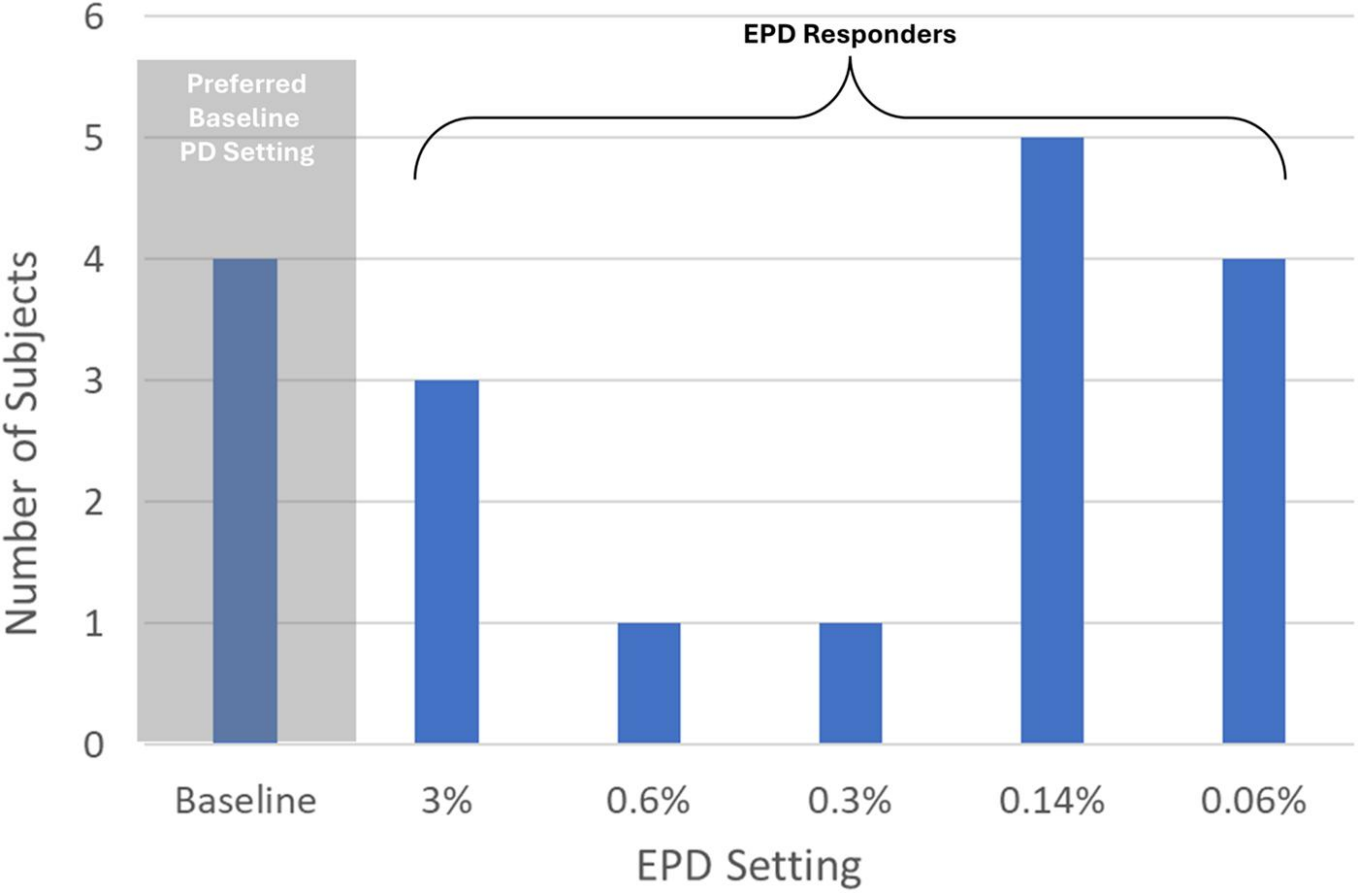
Distribution of lowest preferred pulse dose setting in 18 patients. PD, pulse dose; EPD, extreme pulse dose; Responders, subjects who preferred EPD settings ≤3% PD. This figure shows the preferred PD setting (determined during the EPD titration phase) for each subject who completed the study.

Fourteen of 18 patients reported that EPD was as good as or preferable to their baseline 10 kHz SCS treatment, yielding an EPD responder rate of 78% (Figure 3). This response rate was significantly higher than predicted from previous clinical studies (*p* = 0.04; Fisher's Exact Test). The most common lowest preferred PD was 0.14% (*n* = 5; 28%), followed by 0.06% (*n* = 4; 22%) and 3% (*n* = 3; 17%). One patient selected a PD of 0.6% and 1 patient selected a PD of 0.3%.

### 3.3 Pain intensity

**Figure 4:**
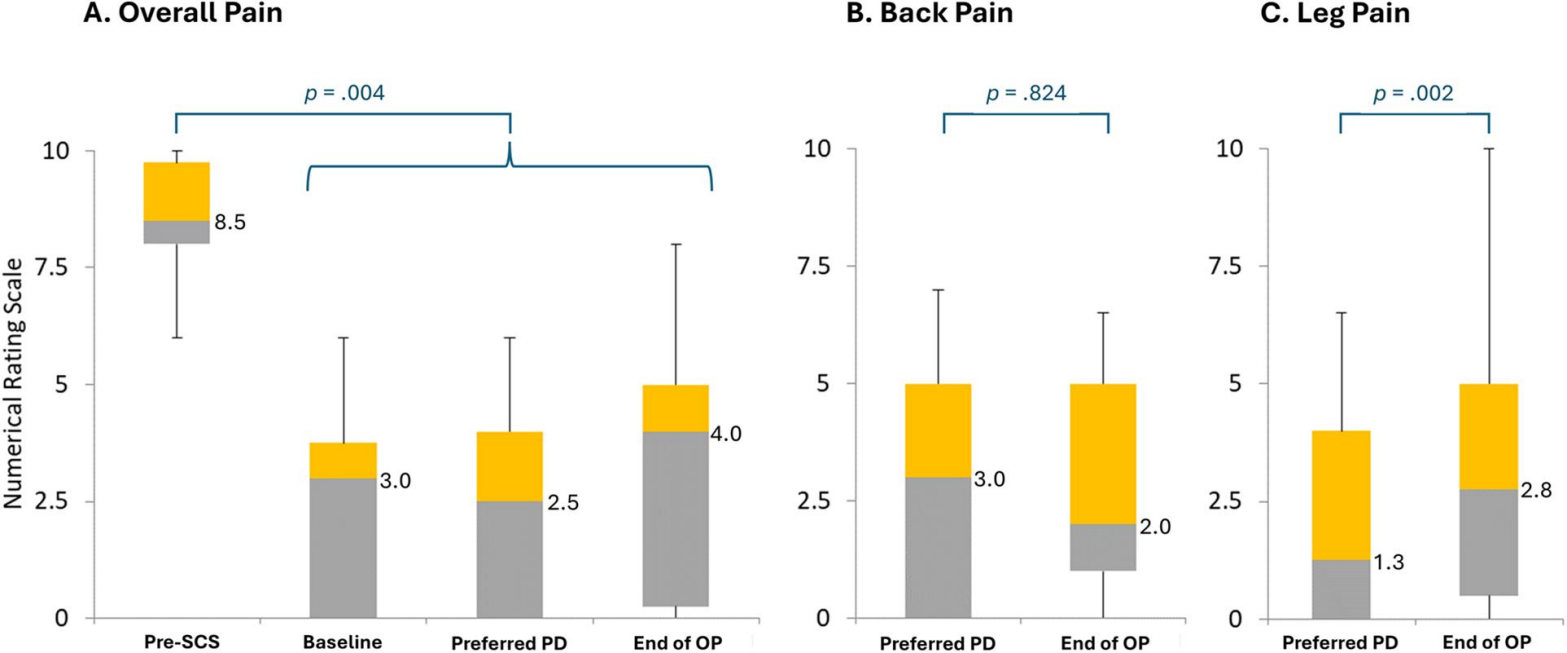
Pain intensity without treatment, at baseline, during an assessment period of the lowest preferred pulse dosing, and at follow-up. End of OP, end of 3-month observation period; PD, pulse dosing; SCS, 10 kHz spinal cord stimulation. **(A)** Overall pain intensity. **(B)** Back pain intensity. **(C)** Leg pain intensity. Preferred PD refers to the EPD titration assessment of the patient's lowest preferred pulse dose setting. Bars represent the distribution of patient scores on the numerical rating scale. Gray bars represent the range from the lowest quartile to the median; orange bars, from the median to the third quartile. Numbers to the right of the bars represent median values. Figure based on diary data collected in all who completed Visit 3 (*n* = 14).

Regarding overall pain, the patient NRS values fell significantly from the pre-SCS period (median, 8.5) compared with the other study periods, including baseline (median, 3.0), during the EPD titration assessment of the lowest preferred PD setting (median, 2.5), and the End of OP (Friedman's Test: median, 4.0; *p* < .004; Figure 4A). For overall pain, NRS values did not vary significantly across timepoints after the pre-SCS period through the End of OP (Friedman-Nemenyi-Q Test: median range, 2.5–4.0; *p* > .05). Regarding back pain, patient NRS values were not significantly higher during the EPD titration assessment of the lowest preferred PD setting (median, 3.0) than at the End of OP (Wilcoxon signed ranks: median, 2.0; *p* = .824; Figure 4B). For leg pain, the patient NRS values did vary significantly between the EPD titration assessment of the lowest preferred PD setting and the End of OP (Wilcoxon signed ranks: median values, 1.3 vs. 2.8; *p* = .002; Figure 4C).

### 3.4 Other patient-reported outcomes

**Figure 5:**
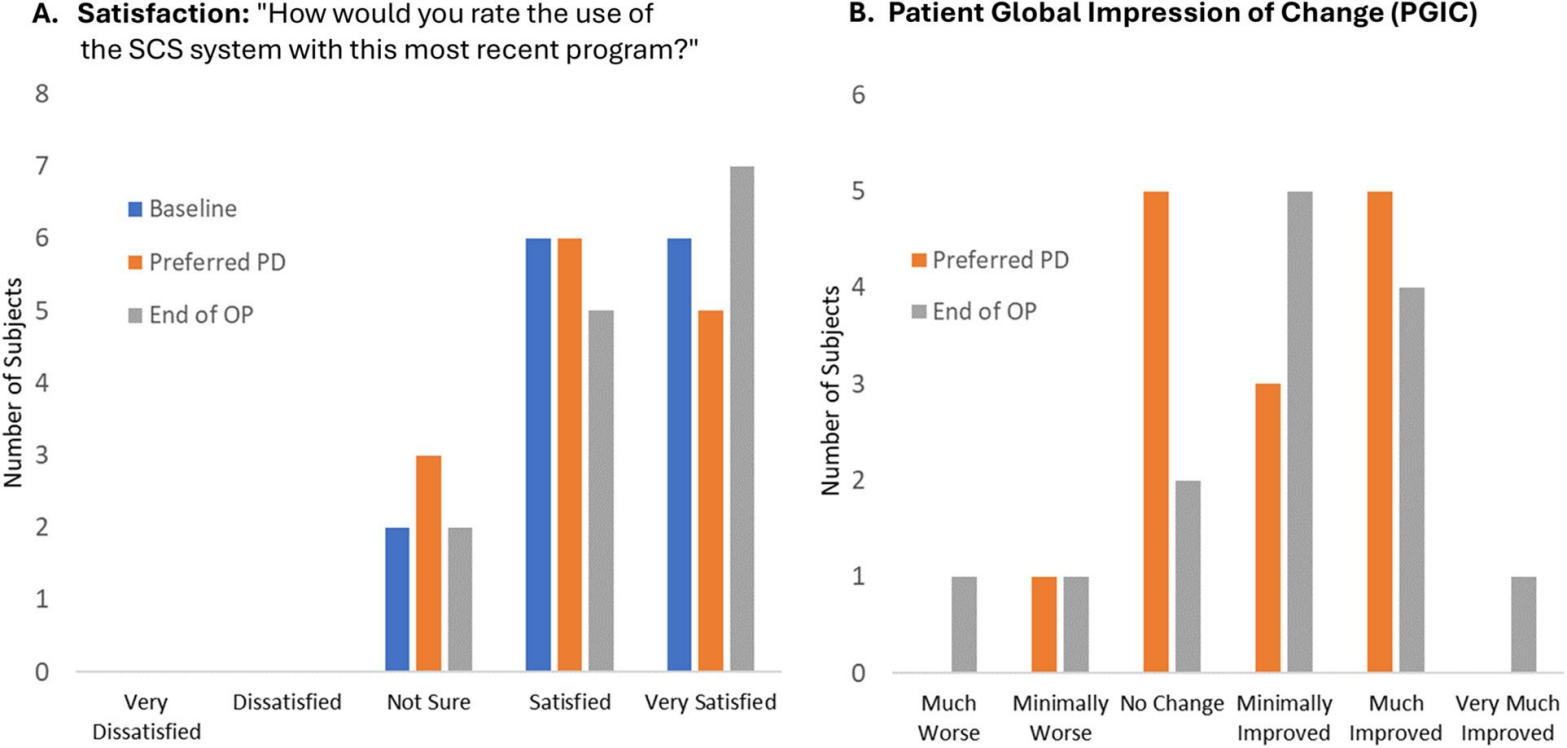
Patient satisfaction and global impression of change. End of OP, end of 3-month observation period; PD, pulse dosing; PGIC, patient global impression of change; SCS, 10 kHz spinal cord stimulation. **(A)** Patient satisfaction with SCS system using most recent program. **(B)** Patient global impression of change from Baseline using most recent program. Lowest PD refers to the EPD titration assessment of the patient's lowest preferred dose setting. Figure data reflect self-reported values recorded in patient diaries at the end of each period.

Most patients reported being satisfied or very satisfied after the EPD titration assessment of their lowest preferred PD setting (11 of 14; 79%) and no patients were dissatisfied with EPD 10 kHz SCS therapy (Figure 5A). Similarly, at End of OP, 12 of 14 patients (86%) were satisfied or very satisfied, and none were dissatisfied. Patient satisfaction did not differ significantly after the EPD titration assessment of their initial lowest preferred PD compared with End of OP (Friedman-Nemenyi-Q Test: *p* > .67). PGIC responses regarding changes in pain compared with baseline were also similar across time periods, with most patients reporting no change or an improvement after the EPD titration assessment of their initial lowest preferred PD setting (13 of 14; 93%) and at End of OP (12 of 14; 92%; PGIC at lowest preferred PD vs. PGIC at End of OP: Wilcoxon signed ranks: *p* = .83; Figure 5B).

**Figure 6:**
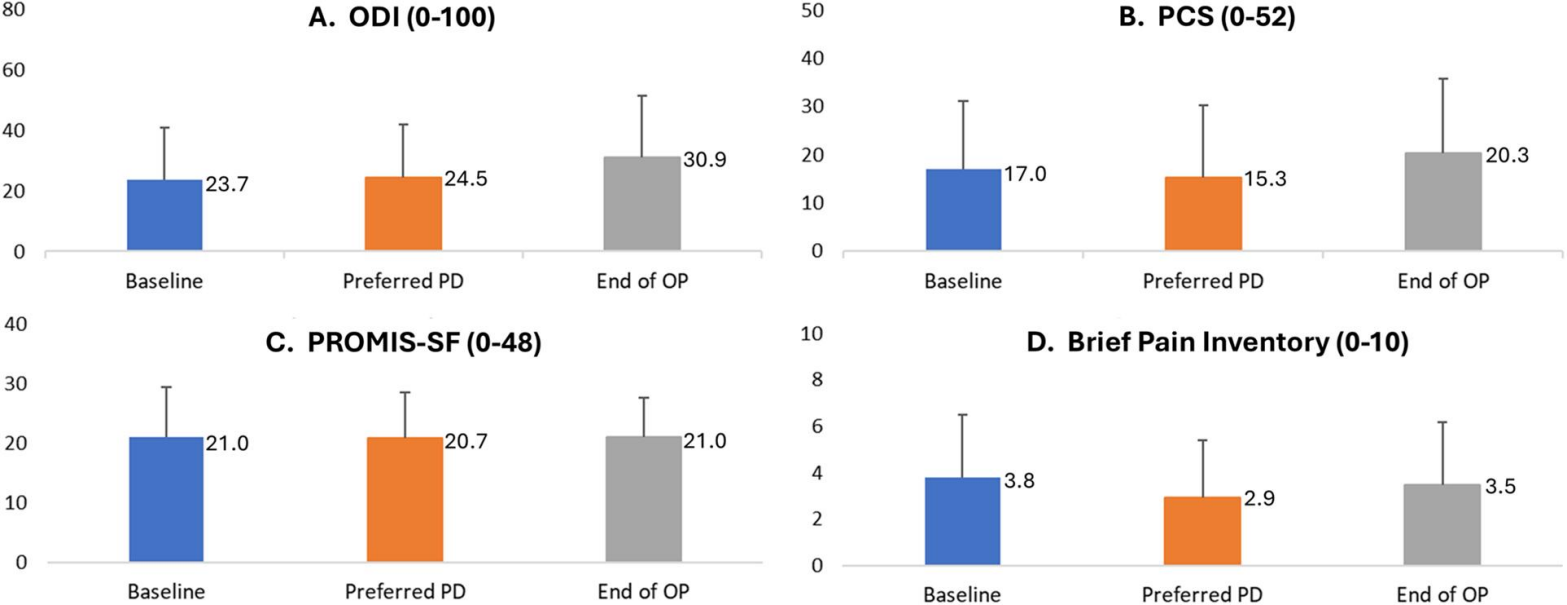
Patient-reported disability, catastrophizing, sleep disturbance, and pain inventory by study period. End of OP, end of 3-month observation period; ODI, Oswestry Disability Index; PCS, Pain Catastrophizing Scale; PD, pulse dose. **(A)** ODI is a self-reported index of disability. **(B)** PCS: Patient Catastrophizing Scale. **(C)** PROMIS-SF is a self-reported index of sleep disturbance. **(D)** Brief Pain Inventory. The bars represent mean scores (with the mean value labelled to the right of each bar), and positive error bars representing standard deviation. Score data were collected in patient diaries at the end of each period. Figure represents information was collected in all patients who completed Visit 3 (*n* = 14). Preferred PD refers to the EPD titration assessment of the patient's lowest preferred pulse dose setting.

As shown in Figure 6, other PROs also remained similar throughout patient follow-up. Mean scores for the ODI, a measure of disability, did not differ significantly across assessments taken at baseline (23.7 ± 17.2), after the EPD titration assessment of the patient's lowest preferred PD setting (24.5 ± 17.2), and at End of OP [30.9 ± 20.5; RM-ANOVA: F(2, 13) = (2.3), *p* = 0.12; Panel A]. Self-reported sleep disturbance also varied little by study period, with mean values of the PROMIS-SF ranging across study periods from 20.8 to 21.0 [RM ANOVA: F(2, 13) = (0.01), *p* = 0.99; Panel C]. Similarly, the impact of pain on the patient's daily life, as measured by mean values of the Brief Pain Inventory, did not vary significantly across baseline (3.8 ± 2.7), after the EPD titration assessment of the patient's initial lowest preferred PD setting (2.9 ± 2.5), and at End of OP [3.5 ± 2.7; RM-ANOVA: F(2, 13) = (0.78), *p* = 0.47; Panel D]. Finally, emotional and cognitive responses to pain, as measured by the PCS, also did not vary across time periods, with mean values of 17.0 at baseline, 15.3 after the EPD titration assessment of the patient's lowest preferred PD setting, and 20.3 at End of OP [RM-ANOVA: F(2, 13) = (0.61), *p* = 0.55; Panel B].

### 3.5 Recharging duration

**Figure 7:**
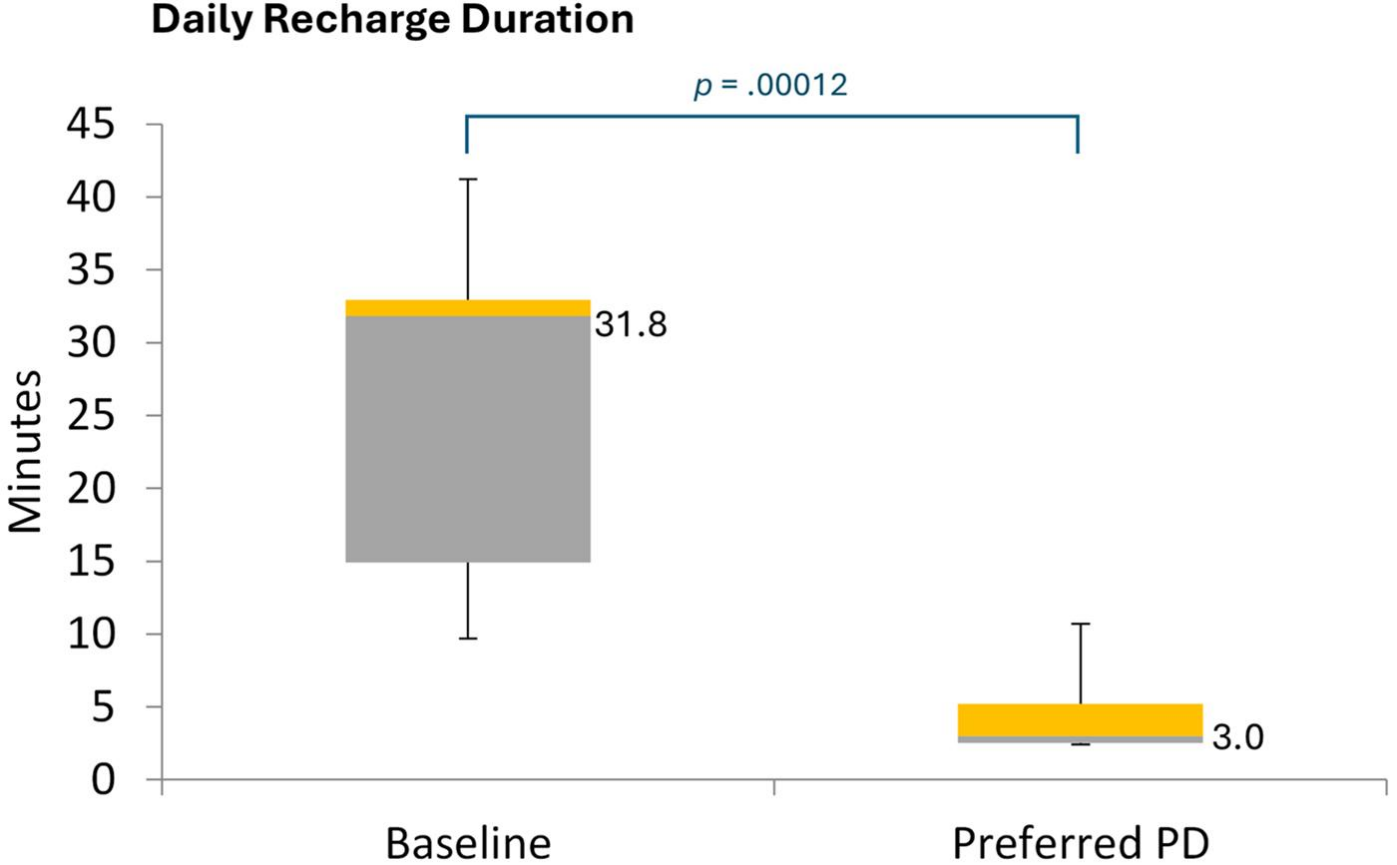
Daily recharge duration. PD, pulse dose. Figure shows the distribution of daily recharge durations for EPD-based 10 kHz SCS, based on diary entries recording over 7 days during each period. The values to the right of the box plots reflect median values.

The median daily time required to recharge the IPG was significantly shorter during the EPD titration assessment of the patient's lowest preferred PD setting than at baseline (median minutes/day, 3.0 vs. 31.8; Wilcoxon signed ranks: *p* = .0001; Figure 7).

### 3.6 Adverse events

Three adverse events were noted during the study: one subject developed wrist, hip, and back pain post-motor vehicle accident; another subject developed new pain where MRI showed progression of spine disease, which was treated with medication, device reprogramming, and facet joint RFA procedure; a third subject contracted COVID, which was treated with medication. None of the adverse events were study-related.

## 4 Discussion

In this prospective case series, we conducted EPD titration for 10 kHz SCS therapy for the treatment of chronic back or leg pain, finding that in 78% of patients, PDs ranging from 3% to 0.06% were as effective in controlling chronic pain as standard 10 kHz SCS over 3 months of follow-up. Notably, our findings showed that EPD had similar effectiveness as standard 10 kHz SCS in relieving both leg and back pain, as well as in achieving good results for PROs assessing patient satisfaction with therapy, global impression of change, sleep disturbance, pain-related impact, and disability.

These findings help expand our understanding of EPD 10 kHz SCS for chronic pain treatment, extending the range of PDs evaluated beyond those of a study by Provenzano et al., which assessed PDs ranging from 3% to 50% (8). Provenzano et al., which to our knowledge is the only other study reporting on PD 10 kHz SCS, found that 81% of patients responded to PD 10 kHz SCS and that outcomes for pain, PGIC, and treatment satisfaction were similar or better than those of standard 10 kHz (8). Taken together, our findings and those of Provenzano et al. suggest that PD 10 kHz SCS, even at very low PDs, may be highly effective in treating chronic back and leg pain. As in the study by Provenzano et al., in the current study, patient follow-up ranged from 11 to 24 weeks and was thus too short to evaluate the long-term durability of EPD. Nevertheless, the trajectory of pain outcomes and PROs were similar to those observed during the first 3 months of trials of standard 10 kHz SCS reporting sustained reductions in pain through 12 and 24 months (3, 17). Thus, our findings provide a positive signal, warranting further investigation of durability of EPD effectiveness in reducing chronic back and leg pain.

While our study findings suggest that EPD 10 kHz may be effective for treating chronic back and leg pain, the mechanisms underlying this approach remain unclear. Electrophysiological data suggest that cycling of SCS stimulation can affect neurocircuitry in ways that support pain suppression (9, 18). For example, burst stimulation, in which “on” and “off” cycles of 500 Hz stimulation are intended to mimic natural neuronal firing patterns, affects multiple neuronal activities in the spinal cord and brain, leading to moderate pain suppression (9, 19). However, clinical data indicate that, to be effective, burst stimulation requires substantially more “On” time than PDs used in the current study (20, 21). Moreover, different SCS modalities appear to rely on different mechanisms of action (18). Notably, 10 kHz SCS exhibits substantially greater selective activation of inhibitory superficial dorsal horn neurons than either burst stimulation or low-frequency SCS highlighting its unique mechanisms of action (5, 18).

Furthermore, PD 10 kHz SCS enables reduced exposure to electrical stimulation; whereas continuous stimulation may promote neuroplasticity changes, fibrosis, and other physiological factors that lead to habituation and reduced long-term efficacy of SCS therapy (22, 23). Indeed, habituation is the most common reason for device explantation, with real-world data showing that the risk of habituation increases with exposure time, reaching over 17% by 5 years after of implant (9, 24). Thus, by reducing the exposure time to SCS stimulation, EPD 10 kHz SCS may reduce the burden of therapy habituation among patients treated with SCS. Furthermore, in a study of patients experiencing loss of efficacy of 10 kHz SCS, a 1-month stimulation holiday led to significantly higher pain relief (23). Thus, by providing a period of very low stimulation, EPD 10 kHz SCS could potentially serve as a rescue therapy for patients experiencing therapy habituation.

Beyond its potential to address therapy habitation, EPD may also increase the convenience of 10 kHz SCS therapy for patients with chronic pain. In the current study, we found that the median IPG recharge time for EPD 10 kHz SCS was only 3 min, which was substantially lower than the 30-minute median value for standard 10 kHz SCS. This finding accords with that of Provenzano et al. who reported a mean recharge times for PD 10 kHz SCS of about 8–26 min compared with 43.8 min for standard 10 kHz SCS (8). Lower recharge times may also extend the longevity of the IPG device, which could help reduce overall treatment costs for 10 kHz SCS therapy (25). However, the actual impact of recharge times on IPG longevity is unknown and will depend on multiple factors, including whether recharging is done on continual basis or all at once (7, 8).

The current study had limitations that merit consideration. The study was conducted in a small and pragmatically selected sample of patients; thus, findings may not be generalizable to all patients eligible for 10 kHz SCS for the treatment of chronic back or leg pain. Nevertheless, mean NRS pain scores taken at pre-SCS (8.6) and baseline (2.4) in this study closely match those reported in trials of 10 kHz SCS for chronic back and leg pain, providing reassurance that our study patients are broadly representative of this patient population (3, 26). The non-blinded nature of the study, though inherent to the intervention, may have influenced patients' responses, potentially biasing study results. Notably, this limitation applies to all studies evaluating SCS therapy for chronic pain treatment. Some patients were already using a PD setting at baseline. However, EPD is more similar to PD than to standard 10 kHz SCS; thus, any bias induced by the baseline use of PD setting is likely to have reduced, not increased, the likelihood of observing that EPD is an effective treatment.

## 5 Conclusion

Using prospectively collected data in a well-defined patient group, this study found that 78% of patients receiving standard 10-kHz SCS continued to experience similar levels of pain relief and quality-of-life outcomes at extremely low PD levels. Given its much lower stimulation output and energy requirements, EPD 10 kHz SCS could substantially lower the risk of therapy habituation, while potentially enhancing patient convenience and treatment costs due to reduced recharge times. Future studies should evaluate the durability of EPD 10-kHz SCS in maintaining pain relief and quality-of-life outcomes over a longer term of follow-up.

## Data Availability

The datasets presented in this article are not readily available because data is proprietary, but data may be available via reasonable request. Requests to access the datasets should be directed to Kerry Bradley, bradley@nevro.com.
